# Posttransplant Tacrolimus-Induced Diabetic Ketoacidosis: Review of the Literature

**DOI:** 10.1155/2018/4606491

**Published:** 2018-05-09

**Authors:** Zaid Ammari, Stella C. Pak, Mohammed Ruzieh, Osama Dasa, Abhinav Tiwari, Juan C. Jaume, Maria A. Alfonso-Jaume

**Affiliations:** ^1^Department of Medicine, College of Medicine and Life Sciences, University of Toledo, Toledo, OH, USA; ^2^Division of Endocrinology, Diabetes and Metabolism, College of Medicine and Life Sciences, University of Toledo, Toledo, OH, USA; ^3^Center for Diabetes and Endocrine Research (CeDER), College of Medicine and Life Sciences, University of Toledo, Toledo, OH, USA; ^4^Division of Nephrology, College of Medicine and Life Sciences, University of Toledo, Toledo, OH, USA

## Abstract

Diabetic ketoacidosis (DKA) in patients receiving tacrolimus as part of their immunosuppressive regimen is a rarely reported adverse event. We report a patient with autosomal dominant polycystic kidney disease (ADPKD) and no known history of diabetes mellitus who presented with DKA, 3 months after kidney transplantation.

## 1. Introduction

For 2 decades now, kidney allograft survival has been shortening despite an obvious decrease in acute allograft rejection. The possibility of a single agent capable of both outcomes is being considered. Tacrolimus, the most potent calcineurin inhibitor, may be the reason for both. Its popularity is clearly a consequence of the excellent short term outcome. However, as second kidney transplant becomes the norm because of the reduced allograft survival, alternative immunosuppressive regimens ought to be considered.

New-onset diabetes mellitus after transplantation (NODAT) is now a well-established adverse effect of calcineurin inhibitors, mostly tacrolimus. NODAT has been reported to occur in 32% of patients after solid organ transplantation and may be the most important contributing factor for decreased long-term allograft survival [1]. Immunosuppressant accounts for 74% of the occurrence of NODAT [2], with a higher incidence in patients receiving tacrolimus than cyclosporine (16.6–33.6% versus 9.8–26%) [3, 4]. Failure to identify and manage glucose homeostasis in a timely manner in these patients lead to a life-threatening complication, DKA.

The case presented here describes an accelerated development of tacrolimus-induced DKA 3 months after kidney transplantation. To our knowledge, only 14 cases of tacrolimus-induced DKA have been reported.

## 2. Case Description

A 44-year-old Caucasian male, with no past medical history of diabetes mellitus, was admitted to the hospital with DKA, three months after receiving a deceased-donor kidney transplant for end stage renal disease (ESRD) secondary to ADPKD. The posttransplant course was unremarkable. Patient's immunosuppressive regimen included tacrolimus 1.5 mg BID, mycophenolate sodium 720 mg BID, and low dose prednisone of 5 mg daily. Patient presented to the emergency department with nausea, polyuria, and abdominal pain. He did not have family history of diabetes mellitus. Physical exam was unremarkable except for mild overweight, body mass index of 27 kg/m^2^. Laboratory work-up revealed hyperglycemia, high anion gap metabolic acidosis, significant ketosis with a beta-hydroxybutyrate level of 4.45 mmol/l (reference range 0.02–0.27 mmol/l), ketonuria, and normal lactate levels. Glycated hemoglobin (A1C) was 9.8% compared to 4.8%, 30 days after transplant. Tacrolimus trough level was 13.9 ng/ml. Glutamic acid decarboxylase (GAD-65) autoantibodies were negative. Infectious etiology for hyperglycemia was ruled out.

The patient received intravenous fluids and a bolus of intravenous insulin followed by continuous insulin infusion which was gradually switched to subcutaneous insulin. Daily insulin requirements were approximately 40 units. He was educated about his new diagnosis and discharged on diabetic diet and subcutaneous insulin therapy. Upon follow-up, tacrolimus dose was adjusted to a lower therapeutic index. Insulin requirements markedly decreased and patient was able to be taken off insulin 9 months after. Glycated hemoglobin (A1C) checked at 9 months was 5.2%.

## 3. Discussion

Many of the risk factors that predispose nontransplant patients to diabetes mellitus have been identified as risk factors for NODAT. Some risk factors are unique to the transplant population. Immunosuppressive agents that contribute to NODAT include glucocorticoids, calcineurin inhibitors, and mTOR inhibitors. Both cyclosporine and tacrolimus increase the risk of NODAT. Tacrolimus is more diabetogenic than cyclosporine [3, 4]. Other risk factors are hepatitis C virus and cytomegalovirus infections, impaired glucose tolerance, perioperative hyperglycemia, HLA matching and donor characteristics, and hypomagnesemia [1, 2, 5]. Interestingly, ADPKD, the cause of ESRD in the present case may confer an increased risk of NODAT [6].

In a study using data from the United States Renal Data System (USRDS), 21,489 patients were enrolled, of whom 4,105 developed NODAT by 3 years after transplant. Diabetes complications developed in 58.3% of patients. DKA developed in 8.1% of patients with NODAT [7]. In most of these cases exposure to high dose steroids (steroid-induced diabetes) appears to be a determining factor. Different from many other protocols, our transplant protocol includes a very short (3 days) exposure to high dose steroids.

Including our case, there are 15 cases of tacrolimus associated DKA presentation in organ transplant patients reported in the literature [8–18]. Summary of these cases focused on clinical presentation and management is described in Table 1. Out of the 15 cases, 6 had kidney transplant [8, 10, 13, 16, 17], 6 had liver transplant [9, 12, 13, 18], 2 had heart transplant [11, 14], and 1 had bone marrow transplant [15]. The mean age of patients was 29.9 ± 15.2 years with no gender predominance (8 females and 7 males). None of the patients had history of diabetes mellitus prior to the transplant. 40% of patients, including our patient presented with DKA within the first 3 months after transplant, with median of 7 months.

Higher body mass index (BMI) has been associated with increased risk for NODAT [2]. However, lower BMI has been reported with tacrolimus-associated DKA in organ transplant patients, with mean of 22.1 ± 4.7 kg/m^2^ as in our case.

Female gender, African American ethnicity, recipients of deceased donor kidney transplant, younger age (33–44 versus >55 years), and recent transplant patients had significantly higher risk of DKA after kidney transplantation [19].

Maintenance immunosuppressive therapy is essential to prevent rejection in renal transplant recipients. Calcineurin inhibitors play an integral role in immunosuppressive regimens, with tacrolimus being the preferred agent over cyclosporine, as several studies showed lower incidence of acute rejections with its use [4, 20]. In addition to lower rates of acute rejections, tacrolimus is better tolerated and preferred by patients compared with cyclosporine. Moreover, tacrolimus does not lower mycophenolate levels unlike cyclosporine and, therefore, relatively lower doses of mycophenolate are needed when tacrolimus is used.

Transplant patients on tacrolimus as part of their immunosuppressive regimen had increased risk of DKA compared to cyclosporine based immunosuppressive regimens [7, 19]. Both calcineurin inhibitors cause toxicity to pancreatic islet beta cells and may directly affect transcriptional regulation of insulin expression [21, 22]. Some evidence suggests however that tacrolimus causes more severe swelling-vacuolization, endoplasmic reticulum stress, and apoptosis of pancreatic islet beta cells [23]. Toxic levels of tacrolimus and higher steroid doses potentiate each other's diabetogenic effects [24]. Tacrolimus's diabetogenic effects therefore threaten the health and longevity of the allograft by predisposing the recipients to microvascular and macrovascular diabetes complications which consequently reduce allograft survival.

Decreased insulin requirement after DKA is suggestive of transient pancreatic damage by toxic levels of tacrolimus which is usually dose dependent and appears reversible [24]. Both tapering tacrolimus regimens and cyclosporine substitution for tacrolimus have been associated with decreased insulin requirements. There is one case report in which everolimus substitution for tacrolimus provided sufficient decline in insulin requirements [17].

Importantly, DKA in renal transplant patients has been associated with increased mortality [19].

## 4. Conclusion

Tacrolimus remains the preferred immunosuppressive agent after kidney transplantation given lower incidence of acute rejections and better patients' tolerance. However, tacrolimus's contribution to new-onset diabetes ketoacidosis, as a consequence of pancreatic islet beta cell toxicity, adds to the accumulating evidence of reduced allograft survival observed since its introduction as the immunosuppressant of choice. Despite rarity of reported cases of posttransplant tacrolimus-induced DKA, it seems possible that the decrease in allograft survival observed in the last two decades is just the consequence of tacrolimus-induced diabetes and its complications. The successful decrease in acute allograft rejection provided by tacrolimus has likely confounded this observation. The development of diabetes mellitus with ketoacidosis in patients on therapeutic tacrolimus levels, with no risk factors for diabetes, highlights the need for alternative immunosuppressive agents that will not compromise patients' allografts long-term survival at the expense of inducing a devastating chronic disease. This case study highlights the importance of regular monitoring of fasting blood glucose in transplant patients on tacrolimus based regimen for early detection of NODAT in order to prevent life-threatening complications. It is also another call for attention on the toxic effects of this potent calcineurin inhibitor.

## Figures and Tables

**Table 1:** Comparison of different characteristics of transplant recipients with tacrolimus-induced diabetes ketoacidosis [8–18] and our case.

	Age (years), Gender	Organ transplant	BMI (kg/m2)	Duration since transplantation (month)	Maintenance immunosuppressant regimen	Presentation	Glucose (mg/dl)/pH/HCO_3_^−^ (mmol/l)	HbA_1_C (%)	Glucosuria, ketonuria, proteinuria	IA-2 Ab/GAD-65 Ab	Tacrolimus level (ng/ml)	Management	Discharge regimen/outcome
*Our case*	*44 M*	*Kidney*	*27.0*	*3*	*TAC + PDN + MPS*	*Nausea, polyuria, abdominal pain*	*493/7.32/15*	*9.8*	*+/+/−*	*NA/−*	*13.9*	*IV saline and insulin* *Tapering TAC regimen*	*SC insulin* *Off insulin in 9 months*

Cho et al.	35 F	Kidney	21.8	6	TAC + PDL + MMF	Polydipsia, dry mouth, weight loss, anorexia, fatigue, confusion	712/6.80/1.4	14.7	NA/+/NA	NA/*−*	11.1	IV saline and insulin CYC substituted for TAC	Diabetic diet

Dehghani et al.	13 F	Liver	NA	7	TAC + MMF	Anorexia, fatigue, dizziness, ascites	742/7.22/10	NA	+/+/NA	NA/NA	16.2	IV saline and insulin	Inpatient death secondary to bacterial sepsis

Dehghani et al.	14 M	Liver	NA	3	TAC + PDL	Nausea, vomiting, fever	390/7.26/10	NA	+/+/NA	NA/NA	14.8	IV saline and insulin	SC insulin

Dehghani et al.	14 M	Liver	NA	4	TAC + MMF	Abdominal pain, fever	432/7.21/12.2	NA	NA/+/NA	NA/NA	16.5	IV saline and insulin	SC insulin

Ersoy et al.	42 F	Kidney	29.8	36	TAC + PDL + AZT	Polyuria, polydipsia, confusion, fatigue	520/7.16/7.9	11.6	+/+/+	NA/NA	30	IV saline and insulin CYC substituted for TAC MMF substituted for AZT	SC insulin Switched to OHA in 6 months

Im et al.	22 F	Heart	22.4	7	TAC	Polydipsia, anorexia, abdominal pain	702/6.9/4	12.1	+/+/NA	NA/NA	>30	IV saline and insulin Tapering TAC regimen	SC insulin Switched to OHA in 3 months

Keshavarz et al.	14 F	Liver	NA	12	TAC + PDN	Chest pain, dyspnea	980/7.08/11	10.5	+/+/+	NA/NA	24	IV saline and insulin CYC substituted for TAC	SC insulin

Masood et al.	17 M	Kidney	NA	12	TAC + PDL + MMF	Polyuria, nocturia, dry mouth, anorexia, vomiting, confusion	702/7.10/6	NA	NA/+/NA	*−*/*−*	NA	IV saline and insulin CYC substituted for TAC	SC insulin

Masood et al.	55 F	Liver	NA	24	TAC + PDL + MMF	Polyuria, dizziness	474/NA/16.4	8.9	NA/+/NA	NA/NA	NA	IV saline and insulin	SC insulin

Öztürk et al.	17 M	Heart	15.4	3	TAC + PDL + MMF	Dyspnea, fatigue	574/7.22/13.3	9.7	NA/+/NA	*−*/*−*	45.4	IV saline and insulin Tapering TAC regimen	SC insulin

Solmaz et al.	24 F	Bone marrow	20.8	2	TAC	Loss of consciousness	890/6.9/4	9.1	+/+/NA	NA/NA	NR	IV saline and insulin CYC substituted for TAC	NA

Toyonaga et al.	43 M	Kidney	18.2	12	TAC + MPL	Polyuria, polydipsia, fatigue, weight loss	925/7.34/23.8	11.8	+/+/+	*−*/*−*	9.4	IV saline and insulin Tapering TAC regimen	Diabetic diet

Tuğcu et al.	44 M	Kidney	NA	1	TAC + PDL + MMF	Polyuria, polydipsia, weakness	862/7.27/15	10.7	+/+/NA	*−*/*−*	9.4	IV saline and insulin Everolimus substituted TAC	SC insulin

Yoshida et al.	50 F	Liver	NA	9	TAC + PDN + AZT	Polyuria, polydipsia, visual blurring	1227/6.93/3	NA	NA/+/NA	NA/NA	21.2	IV saline and insulin	SC insulin

*Abbreviations*. BMI, body mass index; HgbA1C, hemoglobin A1C; IA-2 Ab, islet antigen 2 antibody; GAD-65 Ab, glutamic acid decarboxylase antibody; M, male; F, female; NA, not available; TAC, tacrolimus; PDN, prednisone; MPS, mycophenolate sodium; PDL, prednisolone; MMF, mycophenolate mofetil; AZT, azathioprine; MPL, methylprednisolone; CYC, cyclosporine; NR, normal range; IV, intravenous; SC, subcutaneous; OHA, oral hypoglycemic agents.

## Abbreviations

DKA: Diabetic ketoacidosis
ADPKD: Autosomal dominant polycystic kidney disease
NODAT: New-onset diabetes mellitus after transplantation
ESRD: End stage renal disease.
